# A comparative analysis of three pharmacovigilance system assessment tools

**DOI:** 10.1371/journal.pone.0327363

**Published:** 2025-07-08

**Authors:** Muhammad Akhtar Abbas Khan, Jude Nwokike, Asim Rauf, Zaheer-Ud-Din Babar

**Affiliations:** 1 Department of Public Health, Health Services Academy, Islamabad, Pakistan; 2 Drug Regulatory Authority of Pakistan, Islamabad, Pakistan; 3 Promoting the Quality of Medicines Program Plus, United States of America Pharmacopeia, Twinbrook Rockville, Maryland, United States of America; 4 College of Pharmacy, QU Health, Qatar University, Doha, Qatar; Universiti Sains Malaysia, MALAYSIA

## Abstract

**Background:**

Three key tools are currently available for assessing pharmacovigilance systems at the national level: the Indicator-Based Pharmacovigilance Assessment Tool (IPAT), the World Health Organization (WHO) Pharmacovigilance Indicators, and the Vigilance Module of the WHO Global Benchmarking Tool (GBT). These instruments are designed to evaluate the functionality and performance of national regulatory authorities within the context of their respective pharmacovigilance systems.

**Objectives:**

This study aims to identify, analyze, and compare the core characteristics and operational features of these pharmacovigilance assessment tools to better understand their scope, application, and limitations.

**Methodology:**

A structured document analysis was conducted on the three identified tools. The content was systematically reviewed, categorized, and synthesized to facilitate a comparative evaluation of its design, focus areas, and assessment criteria.

**Results:**

The analysis revealed that the available tools encompass a broad spectrum of indicators targeting different dimensions of pharmacovigilance systems, such as infrastructure, processes, and outcomes. However, exclusive reliance on a single tool may offer a limited perspective, potentially overlooking critical components of a national pharmacovigilance framework.

**Conclusion:**

This study underscores the heterogeneity of existing pharmacovigilance assessment tools and emphasizes the importance of context-specific adaptation. A tailored approach, involving the strategic selection or integration of tools, is recommended to ensure a comprehensive and accurate evaluation of national pharmacovigilance systems.

## Introduction

Pharmacovigilance (PV) is also called the safety of medicines and helps to avoid further catastrophes [1]. The World Health Organization (WHO) recommends its member countries to devise an effective pharmacovigilance system to deal with the identification, reporting and monitoring of adverse drugs reactions (ADRs) [2]. The pharmacovigilance system is aimed to promote and protect public health by ensuring the availability of essential medicines in the market and reducing the burden of ADRs [3]. A national government is responsible for providing safe, effective, and quality medicines to its citizens. To monitor the safety of medicines, an adverse drug reaction (ADR) reporting system is required to be established [4]. Pharmacovigilance should also be promoted as a priority by the drug regulatory bodies [5]. The assessment of a pharmacovigilance system identifies its weaknesses and assists in remediating and improving the quality and quantity of ADR reports as well as other potentials for the improvement of PV systems [6].

In 2008, WHO and the University of Washington entered into a joint project for the development of a global pharmacovigilance strategy. As a first step of the project, the Uppsala Monitoring Center (UMC) was tasked to conduct the study of pharmacovigilance activities of public sector organizations. A questionnaire designed by the UMC in various languages including English, Spanish and French was administered to key informants in more than 100 countries. Almost 50% of the study participants responded to the questionnaire. The purpose of the project was to document the current pharmacovigilance situation of non-ICH countries and to submit a proposal to the Melinda and Bill Gates Foundation to fund global PV [7]. Wilbur, K. et al conducted a survey of 13 Arabic-speaking countries by utilizing the UMC assessment of Country PV Situation questionnaire. The results showed that most of the country’s PV systems are government-funded and 67% of systems facilitated spontaneous ADR submission [8].

WHO and partners also conducted a consultative process. This included face-to-face meetings of stakeholders and discussion on the proposed draft minimum requirements by the WHO advisory committee. The document outlined the minimum requirements for any national PV system. As a minimum, these requirements ensure that a PV system exists and is functioning properly [9].

To assess the structure, process and impact of PV systems there are three available globally recognized assessment tools, i.e., indicator-based pharmacovigilance assessment tool (IPAT) [10], WHO pharmacovigilance indicators [11] and WHO global benchmarking tool (GBT) vigilance module [4]. More than 50 countries have successfully implemented the IPAT to date [12–24]. WHO pharmacovigilance indicators have been used for the assessment of the pharmacovigilance systems of many countries [6,25–30]. By using the GBT, as of December 2024, WHO has listed 15 regulatory authorities at maturity level-3 and three at maturity level-4 [31]. Twenty-two authorities have been listed as transitional NRAs[32]. Korea’s Ministry of Food and Drug Safety (MFDS) [32], Singapore’s HAS and Swissmedic [33] have achieved the WLA status by maintaining a well-established vigilance function.

Each of the three tools serves to evaluate the functionality of national regulatory authorities (NRAs) in the context of national pharmacovigilance systems [4,10,11]. However, determining which tool is superior for NRA assessment is challenging. Although the quantity of mandatory indicators across all tools remains relatively consistent, distinctions arise in the acquisition of data through different core and complementary/supplementary indicators in the first two tools.

The present study aims to contribute to the advancement of pharmacovigilance systems through a systematic analysis and comparison of existing assessment tools. The primary objective of this study is to identify, evaluate and compare the characteristics and functionalities of different pharmacovigilance assessment tools. These tools are developed to assess the performance of pharmacovigilance systems worldwide.

## Methodology

To achieve the objectives of the study, document analysis was conducted on the identified three tools, i.e., indicator based pharmacovigilance assessment tool (IPAT) [10], WHO pharmacovigilance indicators [11] and the vigilance module of the WHO global benchmarking tool (GBT) [4]. Document analysis was selected as it facilitates answering research questions by providing valuable insights and information that can shape the understanding of a topic or issue [34]. A similar approach was used in other studies as well [35–38]. The findings of the document analysis were summarized and organized to facilitate comparison and evaluation. The document analysis provided valuable insights into the purpose, objectives, and specific aspects of pharmacovigilance that the IPAT, WHO pv indicators and vigilance module of the WHO global benchmarking tool.

USAID’s indicator-based pharmacovigilance assessment tool, published in 2009, is commonly used to assess the function, capacity, and gaps of the current PV system. The assessment includes a document review, structured interviews using assessment questions, key informant interviews, and additional data from respondents. IPAT contains 43 indicators, out of which 26 are core indicators and 17 are supplementary. These indicators address five pharmacovigilance and medicine safety system components namely policy, law, and -regulation; systems, structures, and stakeholder coordination; signal generation and data management; risk assessment and evaluation; and risk management and communication. The indicators are further classified by “structure,” “process,” or “outcome” according to the product or result they measure. National regulatory authorities, public health programs (PHPs), hospitals and health facilities, the pharmaceutical industry and other concerned can use IPAT [10]. IPAT was the first to identify the need for active surveillance as part of risk evaluation in low- and middle-income countries.

The WHO published an indicator-based manual for the assessment of the PV system in 2015. The WHO has set up minimum requirements for a functional PV center and proposed indicators are based on these requirements. The manual does not replace the World Health Organization’s global benchmarking tool for NRAs. The manual provides indicators for the assessment of national regulatory authority and public health programs. There is a total of 63 indicators. Out of that 27 are core PV indicators including 10 structural, 9 processes, and 8 outcome or impact indicators, and 36 complimentary indicators comprise 11 structural, 13 processes, and 12 outcome or impact indicators. There are nine separate indicators for public health programs [11].

WHO uses a global benchmarking tool for the evaluation of the national regulatory system of medicines. A mapping of internal and external benchmarking tools led to the development of the unified WHO GBT in 2013. Vigilance is one of the modules of the GBT [4]. It contains 6 main indicators and 26 sub-indicators. There is no grouping of core or complementary/supplementary indicators. NRA’s assessment tool includes a subset of indicators from the WHO manual of pharmacovigilance indicators to assist in assessing pharmacovigilance as a delivery item of the NRA [4].

Pre-existing text and data from the three selected pharmacovigilance (PV) assessment tools were systematically collected, reviewed, and analyzed. These tools were examined with the aim of extracting, synthesizing, and comparing key indicators relevant to national PV systems’ performance and maturity. A comprehensive list of core and supplementary/complementary indicators was compiled and organized into thematic categories (see S1 Table in supporting information). This list was not limited solely to the indicators explicitly mentioned in the selected tools. Rather, it was informed by broader literature, including a recent systematic review that identified 77 health service indicators diverging from the WHO’s standard PV indicators [39]. A qualitative content analysis approach was employed to examine the structure, intent, and thematic focus of each tool. The comparative analysis criteria were developed iteratively through an initial exploratory review of the tools, followed by expert consultation and thematic coding. The extracted data were tabulated and analyzed using Microsoft Word.

All documents were in digital (PDF) format and were reviewed in their most recent available versions as of the time of analysis. The primary author conducted the initial data extraction from the full-text versions of each tool. The senior team members (co-author with regulatory experience in pharmacovigilance system strengthening) independently reviewed the extracted content for accuracy and completeness. The synthesized and categorized data were reviewed and approved by all co-authors, with final responsibility resting with the corresponding author.

## Results

The results of the study revealed a diverse range of pharmacovigilance tools designed to assess different aspects of drug safety (S1 Table in supporting information). These tools ranged from simple reporting platforms to advanced studies on medicine safety. The PV and drug safety system of a country must meet all core indicators in order to be considered fully functioning, according to IPAT. WHO also considers the core indicators as most relevant, important and useful. Accordingly, Table 1 presents the number of core indicators for each component within each tool.

**Table 1:** Number of core indicators for each component within each tool.

	WHO indicators	IPAT indicators	GBT vigilance indicators
Existence of PV center	1 core indicator	1 core indicator	No indicator
Legal provisions regulations and guidelines	1 core indicator	2 core indicates	7 sub-indicators
Existence of a national regulatory authority	1 core indicator	No core indicator	No indicator
Existence of budgetary provisions	1 core indicator	1 core indicator	1 sub-indicator
Human resource, training	1 core indicator	1 core Indicator	4 sub-Indicators
Pharmacovigilance part of curriculum	1 core indicator	No core indicator (1 supplementary indicator)	No indicator or sub-indicator
Existence of ADR reporting form	1 core indicator along with 5 subset indicators	4 core indicators	1 sub-indicator
Collection and assessment of ADRs and AEs.	1 core indicator	1 core indicator	1 sub-indicator
Existence of a national advisory committee	1 core indicator	1 core indicator	2 sub-indicators
Risk assessment and evaluation	9 core indicators	2 core indicators	1 sub-indicator
Signal and data management	8 core indicators	4 core indicators	2 core indicators
Existence of drug information center		1 core indicator	
Existence of procedures, SOPs, protocols		4 core indicators	2 core indicators
Sampling of medicines		1 core indicator	
Communication		2 core indicators	3 sub indicators
Existence of a newsletter	1 core indicator	1 core indicator	1Sub indicator

According to the results, IPAT and WHO pharmacovigilance indicators both require a national pharmacovigilance center. GBT’s vigilance module does not contain a standalone indicator of the requirement for an NPC, but it does require an NPC as part of the national vigilance system. It is crucial for all frameworks to have legal provisions, regulations, and guidelines with varying amounts of core indicators and sub-indicators. Compared with WHO indicators, IPAT and GBT vigilance frameworks tend to cover these aspects more thoroughly. One framework recognizes NRAs as essential, namely WHO indicators. The funding of PV activities is recognized across all frameworks as a key indicator of commitment to drug safety.

Across all frameworks, adequate human resources and training are emphasized, with GBT vigilance presenting additional indicators. According to the WHO framework, PV should be incorporated into educational curricula. IPAT considers it supplementary, while GBT neither consider it core nor supplementary. Adverse drug reactions (ADRs) reporting forms and procedures for collecting and assessing these reactions are widely accepted, though specific indicators and subsets differ.

A national pharmacovigilance advisory committee, formed by NRAs, is required for each tool. Each framework includes a comprehensive risk assessment and evaluation as core indicators, although the number and type of sub-indicators may differ. Data management and signal detection are key components of robust pharmacovigilance practices, with all frameworks documenting core indicators. Drug information Centre is recognized by IPAT, while standardized operating procedures (SOPs) are recognized by IPAT and GBT. A core value of IPAT is the sampling of medicines for testing. Newsletters and other forms of communication are recognized as effective communication strategies by IPAT and GBT frameworks, but specifics differ while WHO indicators only specify the availability of newsletters.

A comparative analysis of pharmacovigilance system assessment tools includes the key components (Table 2).

**Table 2:** A comparative analysis of pharmacovigilance system assessment tools.

Components	IPAT	WHO PV indicators	WHO GBT (Vigilance module)
Scope and purpose of the assessment tool	The focus is on assessing pharmacovigilance systems at national, provincial, PHP and HF levels using predefined indicators and benchmarks. IPAT is suitable for evaluating the current state of collection, analysis, and interpretation of data on the safety aspects of medicine regulation as well as to ensure the safe use of medicines at public health programs, health facilities, and the health care worker and consumer levels.	The purpose of this manual is to serve as a tool for quality assurance and improvement.	An assessment of national pharmacovigilance systems in comparison with global standards and recommendations for improvement.
Methodological framework	Uses predefined indicators and scoring systems to develop a structured assessment process.	This document provides a set of indicators as well as guidelines for data collection and analysis.	The comprehensive framework (Structure/Foundation/Input/ Process/ Output ) has standardized criteria for evaluating pharmacovigilance systems.
Indicator analysis	The indicators are categorized into five components, categorized into structural, process and outcome indicators. Further classified into core and supplementary indicators. Indicators focus on organizational structure, signal generation and data management, risk assessment and evaluation, risk management and communication A country must achieve all of the core indicators to be considered to have a minimally functional pharmacovigilance and medicine safety system.	Offers specific indicators classified into structural, process and outcome/impact indicators. Further categorized into core and complementary indicators. It focuses on organizational structure, measuring pharmacovigilance activities, and measuring the ability of the pharmacovigilance system, regulatory actions, and communication activities.	Utilizes a wide range of indicators covering regulatory framework, effective organization and good governance, human resources, procedures in place, monitoring regulatory performance and output, communication, and signal management.
Comparative metrics	A scoring system is provided for each indicator to allow for the comparison of different systems of pharmacovigilance.	There is no scoring system to quantify the indices. The tool allows for the comparison of performance based on standardized indicator values and benchmarks.	A rating scale allows global standards and national pharmacovigilance systems to be compared, identifying performance gaps.
Data collection and management	It often involves utilizing existing pharmacovigilance databases and reports to collect data for predefined indicators.	Focuses on quality and completeness while encouraging systematic data collection and management.	The WHO GBT vigilance module requires systematic data collection and reporting, which usually involves coordinated efforts with national regulators.
Usability and implementation	The guidelines provided are designed to be user-friendly, enabling pharmacovigilance stakeholders to effectively implement assessment and scoring processes.	The provided indicators and guidelines are standardized, aiming to facilitate ease of use and implementation by national pharmacovigilance centers.	To objectively assess medical and vaccine regulatory capacity, developed for national regulatory authorities which involves capacity building and technical assistance.
Strengths and limitations	Strengths include standardized assessment, comparability, and identification of areas for improvement. Limitations may include •Scoring may be subject to subjectivity based on self-reported data. •These indicators have not been tested for sensitivity and specificity. •The indicators do not include those related to non-medication-related patient safety.	Strengths include global standardization, comparability, and emphasis on data quality. Limitations may include resource constraints for data collection and analysis. Limitations for each indicator are provided.	Strengths include comprehensive evaluation, global benchmarking, and capacity building. Limitations may include resource-intensive implementation and dependency on national regulatory capacity. Limitations for each indicator are provided.
Regulatory Context	Based on local needs and priorities, the application can be adapted to different regulatory contexts.	It is designed for regulatory authorities to monitor pharmacovigilance activities and ensure compliance with global standards.	Designed to help low and middle-income countries build regulatory capacity and strengthen their regulatory systems at the national level.

Supplementary or complementary indicators for IPAT and WHO indicators are described in S2 Table in supporting information, while indicators for PHPs are detailed in S3 Table in supporting information.

## Discussion

The three commonly used tools to evaluate NRAs in the context of national PV systems share a common goal to assess NRA functionality. The choice of tool depends on the specific needs and objectives of the NRA evaluation, as well as the context and available data. Various studies in low-and middle-income countries identified that medicines safety issues are linked to weak ADR reporting systems [40,41]. An absent or dysfunctional PV system causes regulatory actions to fail and medicine safety to be compromised [30].

A National Pharmacovigilance Center (NPC) is required by both IPAT and WHO pharmacovigilance indicators. GBT vigilance module does not separately provide an indicator of the requirement of an NPC, but it does require that the national vigilance system includes an NPC. An NPC of a member country of the WHO Programme for International Drug Monitoring (PIDM) is responsible for sharing reports with VigiBase [42]. It is the basis for national PV systems since every country is recommended to establish an NPC by the WHO [43].

Embedding pharmacovigilance into the curriculum of health professionals plays a critical role in preparing them for career challenges on issues of medicine safety [44], which is a core indicator of the WHO indicators. On the IPAT, pharmacovigilance a part of the curriculum of HCPs is considered a supplementary indicator, and forms a part of the assessment of the national pharmacovigilance system. It is worth mentioning that the vigilance module of GBT does not ask questions on the inclusion of PV into HCPs curriculum while evaluating the NRA. Healthcare providers have a role to play in increasing vaccine awareness in the population, according to a study [45]. A study argued that developing countries lack professionals who are capable of evaluating drug safety and managing risks [46]. Future pharmacists and healthcare professionals should be trained in PV and ADR reporting in order to instil a culture and practices associated with ADR reporting during their professional careers [46–48]. Some studies, by using the WHO PV indicators, evaluated the national PV systems by incorporating questions on PV in the HCPs curriculum [6,25,29,30].

The WHO pharmacovigilance indicators provide eight outcomes or impact indicators that measure the effectiveness of pharmacovigilance systems in different countries. These indicators require quantitative data. However, in low and middle-income countries (LMICs), the collection of data from hospitals can be challenging, hence it becomes difficult to collect specific data on the number of adverse drug reactions (ADRs)-related hospital admissions and deaths [49,50]. A study in Nigeria concluded that it was difficult to compute the process and outcomes indicators because of poor record keeping in all facilities [27] on LMICs utilizing the WHO indicators for pharmacovigilance may not have collected information for these indicators due to the difficulty of obtaining accurate and reliable data. On the other hand, it is relatively easy to collect information about how many signals have been sent and what regulatory actions have been taken by the NRA. This may explain why epidemiological data is less frequently required by WHO indicators. IPAT, on the other hand, does not require similar data, which makes it simpler to use.

One of the major challenges in calculating the costs of treatment of medicines-related illnesses, costs of hospitalization, and extension in hospital stay in LMICs is the lack of robust monitoring systems and mechanism to capture relevant data. A combination of advanced methodologies such as machine learning and the availability of large amounts of electronic healthcare data offers the potential for optimizing drug benefit-risk profiles in real-world environments [51]. A systematic review found that PV system performance was low in terms of both ‘process’ and ‘outcome’ indicators, which reflected the incapacity to collect and use local data to identify signs of drug-related problems [29].

Despite not being recognized by other tools, IPAT recognizes the drug information centre as an independent entity. By providing education on medicines and supporting pharmaceutical services, medicine information services can also help minimize medication errors [52]. The IPAT indicator assesses the quality of medicines in circulation in a particular country. Both WHO indicators and GBT lack this indicator. A sampling and testing program allow the government to confirm that the products on the market are compliant with its specifications [53].

The Erice declaration emphasizes the importance of communication on drug safety [54]. In order to ensure medicine safety, it is important to communicate risks related to real and potential safety issues [55]. Only one core indicator is provided by the WHO indicators in terms of core communication requirements, and that is the existence of a newsletter. In addition, a complementary indicator asks whether the pharmacovigilance center has communication facilities. The same indicator on IPAT is categorized as a core requirement.

It is the complementary indicators (S2 Table in supporting information) of WHO PV indicators that make the real difference with IPAT. For many LMICs, collecting data on complementary indicators is difficult because most of the studies only utilized WHO core indicators [6,26,27,30,56]. Data on medicines consumption and prescription, HCP’s knowledge of ADRs, congenital malformations caused by medicines, and average work or school days lost due to drug problems are complementary indicators. The cost savings associated with pharmacovigilance activities, the health budget impact associated with pharmacovigilance activities, the knowledge of patients about ADRs and the rationale for the use of medicines are all specific to WHO indicators. [27].

WHO PV indicators provide only nine pharmacovigilance indicators for assessing public health programs (PHPs), whereas IPAT provides 31. There are seven indicators on both tools which completely or partially seek similar information, while the others ask for different details. Researchers can use both tools separately to evaluate PHPs and obtain better insights by combining them. Currently, one study has combined the WHO indicators for PV and IPAT to assess the PHPs. Barry et al conducted a comparative assessment of the PV systems within the neglected tropical diseases programs in four East African countries including Tanzania, Kenya, Ethiopia, and. Rwanda. The study shows that the pharmacovigilance system in public health programs is neglected in EAST African countries. The authors utilized “the East African community harmonized PV indicators tool for PHPs” to interview the staff of the national NTD programs. This tool was derived from the WHO pharmacovigilance indicators and the IPAT [56]. Another study [57] also utilized the WHO-based indicators to assess the pharmacovigilance activities in the national HIV/AIDS, malaria, and tuberculosis control programs. However, the majority of the studies that evaluated the PHPs as part of national PV systems have utilized the IPAT [12–15,23,58].

In particular, two indicators on IPAT, namely, “number of medicine utilization reviews conducted in the last year and number of active surveillance activities currently ongoing or carried out in the last five years”, and two indicators on WHO manual, namely, “Number of medicine-related hospital admissions per 1000 individuals exposed to medicines in the public health programme in the previous year and Number of medicine-related deaths per 1000 individuals exposed to medicines in the public health programme in the previous year”, require studies that are cost and time-consuming. However, these studies are vital for introducing priority interventions to improve the PV system in PHPs.

Like IPAT, WHO’s PV indicators manual does not provide a separate module for the assessment of hospitals. However, several indicators in WHO’s tool can be selected for the assessment of hospitals. Opadeyi *et al* studied the status of PV in tertiary care hospitals in the South-South Zone of Nigeria by administering the modified WHO indicator-based tool [27].

### Recommendations

Pharmacovigilance is an evolving field, and none of these tools is able to capture all emerging issues. For instance, treatment modalities and medical practices are becoming more innovative and complex. We recommend revising the pharmacovigilance assessment tools to take into account these new areas. To meet the changing regulatory landscape, PV tools must also be fit for purpose. For instance, periodic revision of PV tools could help to cover topics related to facilitated regulatory pathways (FRP) that ensure their safety surveillance systems with the capacity to provide additional data post-approval. Developing and harmonizing a fit for all pharmacovigilance tools can assist policymakers and regulators with gap assessments, interventions, and improving service delivery by healthcare providers. Such a tool will enable a more efficient and standardized approach to monitoring and improving drug safety across diverse populations and healthcare systems.

Social media is a popular and free way to disseminate information. Studies suggested that the PV system could be strengthened by the use of social media [59,60]. A PV assessment tool may include social media use in medicine safety communication by NRAs as a core or supplementary indicator.

Two indicators 4.6 and 4.7 in the IPAT framework (“percentage of patients in public health programs for whom drug-related adverse events were reported in the last year” and “percentage of patients undergoing treatment within a public health program whose treatment was modified because of treatment failure or ADRs in the last year”) are duplicated in both modules assessing ministry of health or NRA and public health programs. The scope of indicator 4.7 appears to focus more on the assessment of the public health program rather than the NRA. In light of this, it would be appropriate to place these indicators in the PHP module.

Also, there is a need to enhance active surveillance capacity to support the introduction of new medicines and to utilize the increasing availability of electronic medical records and pregnancy exposure registries. Artificial intelligence enhances the accuracy, efficiency, and completeness of drug safety monitoring through machine learning algorithms. Innovative and sophisticated treatment modalities, such as biologics, cell and gene therapy, complex generics, and combination products, have gained significant attention in recent years. While these advanced therapies offer promising treatment options, the existing pharmacovigilance indicators may not fully encompass the diverse range of emerging formulations, such as long-acting injectables and implantable drugs [61,62]. It is also recommended that pharmacovigilance indicators should be periodically revised to include emerging areas. For instance, use of RWD/RWE, AI, and facilitated regulatory pathways (FRP) support the capacity to monitor the real-world effectiveness of products that were fast-tracked. Also, there are no indicators that cover manufacturing variations and their impact on the safety and quality of products, and nothing on specifications and field alert reporting from manufacturers. The emergence of complex generics, biologics, drug-device combinations, and cell and gene therapy may also require that more functionalities be included in the pharmacovigilance assessment tools.

### Limitations and study implications

This content analysis study is subject to several limitations. First, the analysis was based solely on publicly available documents and methodological frameworks of the selected pharmacovigilance system assessment tools, which may not fully capture their practical implementation nuances across different regulatory settings. Additionally, the study did not involve stakeholder interviews or field validation, which could have provided contextual insights into how these tools are perceived, adapted, or challenged in real-world applications. The comparison may also reflect an interpretative bias, as the coding and thematic categorization depended on the researchers’ subjective judgment, despite efforts to ensure analytical rigour and transparency.

Despite these limitations, this comparative analysis offers valuable insights for national regulatory authorities and global health partners seeking to strengthen pharmacovigilance systems. It highlights the strengths and gaps across the assessment tools, supporting informed selection or customization of frameworks suited to country-specific needs. Moreover, the findings underscore the importance of integrating contextual adaptability, stakeholder engagement, and continuous feedback mechanisms into system assessments. Future research could build upon this study by incorporating empirical evaluations, including user experiences and outcomes of applying these tools in diverse regulatory environments.

## Conclusion

This study highlights the diversity of pharmacovigilance tools available and the need for customization when selecting the tools to suit the specific requirements of a specific pharmacovigilance program. Pharmacovigilance is an evolving scientific field. It evolves as treatment modalities and medical practice evolves. A joint working group comprising experts mainly from WHO, UMC and regulators from high and low-income countries can be established to reach a harmonized PV tool for assessment of the national pharmacovigilance system. This collaboration aims to have a common PV tool for the assessment of the national PV system and to improve the safety and efficacy of medicine worldwide by sharing information, expertise, and best practices across different regions and economic settings.

## Supporting information

S1 Table Comprehensive list of core and supplementary/complementary indicators. (DOCX)

S2 Table Supplementary or complementary indicators for IPAT and WHO indicators. (DOCX)

S3 Table Indicators for PHPs. (DOCX)
