# Supplementary material for: A comparative analysis of three pharmacovigilance system assessment tools
Source: PLoS One. 2025 Jul 8;20(7):e0327363. doi: 10.1371/journal.pone.0327363 (PMC12237061; doi:10.1371/journal.pone.0327363)
Supplement: S1 Table — (DOCX) [file pone.0327363.s001.docx]

| **S1 Table : Comprehensive list of core and supplementary/complementary indicators** | | |
| --- | --- | --- |
| **(Indicator Code) WHO core indicators** | **(Indicator No.) IPAT core indicators** | **GBT Indicators** |
|  | **Core Structural indicators** |  |
| **Existence of PV Center** |  |  |
| CST1. Existence of a pharmacovigilance centre, department or unit with a standard accommodation | 2.1. Existence of a pharmacovigilance center or unit |  |
| **Legal Provisions Regulations and Guidelines** |  |  |
| CST2. Existence of a statutory provision (national policy, legislation) for pharmacovigilance | - 1. Existence of a policy document that contains essential statements on pharmacovigilance or medicine safety (stand alone or as a part of some other policy document)   2. Existence of specific legal provisions for pharmacovigilance in the national medicines legislation or similar legislation   2.2 Pharmacovigilance center or unit has a clear mandate, structure, roles, and responsibilities | VL01.01: Legal provisions for a national vigilance system exist.  VL01.02. Legal provisions and regulations require the manufacturers and/or MAHs to set up a vigilance system of their medical products and periodically report vigilance data to the NRA.  VL01.03. Guidelines ensure that distributors, importers, exporters, healthcare institutions, consumers and other stakeholders are encouraged to report adverse drug reactions (ADRs) and AEs to the MAH and/or NRA.  VL01.04: Legal provisions and regulations allow NRA to require manufacturers and/or MAHs to conduct specific studies on safety and effectiveness under specific conditions.  VL01.05. Legal provisions, regulations and guidelines require manufacturers and/or MAHs to designate an individual person to be in charge of vigilance system.  VL01.06. There are guidelines for planning, conducting, monitoring, and reporting of vigilance activities.  VL01.07. Legal provisions and regulations allow recognition and/or reliance on vigilance-related decisions, reports or information from other countries or regional or international bodies. |
| **Existence of a NRA** |  |  |
| CST3. Existence of a medicines regulatory authority or agency |  |  |
| **Existence of Budgetary provisions** |  |  |
| CST4. Existence of any regular financial provision (e.g. statutory budget) for the pharmacovigilance centre | 2.5. Dedicated budget available for pharmacovigilance-related activities |  |
| **Human Resource, Training** |  |  |
| CST5. The pharmacovigilance centre has human resources to carry out its functions properly | 2.4. A designated staff responsible for pharmacovigilance or medicine safety activities | VL03.01: Adequate, competent staff (i.e., education, training, skills and experience) is assigned to perform vigilance activities.  VL03.02: Duties, functions, and responsibilities of the staff in charge of vigilance activities are established and updated in the respective job descriptions  VL03.03: Training plan developed, implemented and updated at least once a year for staff in charge of vigilance activities.  VL03.04: The NRA creates and maintains records of staff training activities and training effectiveness verification. |
| **Pharmacovigilance part of Curriculum** |  |  |
| CST8. Incorporation of pharmacovigilance into the national curriculum of the various health-care professions (includes subset indicators:  CST8a: for medical doctors;  CST8b: for dentists;  CST8c: for pharmacists;  CST8d: for nurses or midwives;  CST8e: for others − to be specified) | 2.12. Percentage of predefined core pharmacovigilance topics present in the preservice training curricula (disaggregated by medicine, pharmacy, nursing, and public health curricula)  Supplementary indicator |  |
| **Existence of a newsletter** |  |  |
| CST9. Existence of a newsletter, information bulletin or website for dissemination of pharmacovigilance information | 2.10. Existence of an ADR or medicine safety bulletin (or any other health-related newsletter that routinely features ADR or medicine safety issues) published in the last six months | VL06.01: Vigilance activities and relevant feedback are appropriately communicated to the public. |
| **Existence of ADR Reporting Form** |  |  |
| CST6. Existence of a standard ADR reporting form in the setting  Subset indicators: The standard reporting form provides for reporting:  CST6a: suspected medication errors;  CST6b: suspected counterfeit/substandard medicines;  CST6c: therapeutic ineffectiveness;  CST6d: suspected misuse, abuse of and/or dependence on medicines;  CST6e: ADRs by members of the general public | 3.3. Existence of a form for reporting suspected ADRs  3.4. Existence of a form for reporting suspected product quality issues (as a subset in the ADR form or as a separate form)  3.5. Existence of a form for reporting suspected medication errors (as a subset in the ADR form or as a separate form)  3.6. Existence of a form for reporting suspected treatment failure (as a subset in the ADR form or as a separate form) | VL04.01: Vigilance procedures and tools are in place and implemented for collection and assessment of ADRs and AEs.  VL04.02: Vigilance procedures and tools are in place for investigation, interpretation of and response to ADRs and AEs. |
| **Collection and assessment of ADRs**  **and AEs.** |  |  |
| CST7. A process is in place for collection, recording and analysis of ADR reports | 3.1. Existence of a system for coordination and collation of pharmacovigilance data from all sources in the country (e.g., health programs, immunization program, active surveillance studies) | VL04.01: Vigilance procedures and tools are in place and implemented for collection and assessment of ADRs and AEs. |
| **Existence of a national Advisory Committee** |  |  |
| CST10. Existence of a national ADR or pharmacovigilance advisory committee or an expert committee in the setting capable of providing advice on medicine safety. | 2.6. Existence of a national medicine safety advisory committee or a subcommittee with similar functions that has met at least once in the last year | VL04.06: The NRA has access to expert committees for review of serious emergent safety concerns, when needed.  VL04.07: With respect to vigilance data, assessment of the risk-benefit balance of medical products is regularly conducted |
| **Risk assessment and evaluation** |  |  |
| CP1. Total number of ADR reports received in the previous calendar year (also expressed as number of ADRs per 100 000 persons in the population) | 4.4. Number of ADR reports received in the last year |  |
| CP2. Current total number of reports in the national, regional or local database |  |  |
| CP3. Percentage of total annual reports acknowledged and/or issued feedback |  |  |
| CP4. Percentage of total reports subjected to causality assessment in the previous calendar year |  |  |
| CP5. Percentage of total annual reports satisfactorily completed and submitted to the national pharmacovigilance centre in the previous calendar year  Subset indicator CP5a: of the reports satisfactorily completed and submitted to the national pharmacovigilance centre, percentage of reports committed to the WHO database |  |  |
| CP6. Percentage of total reports attributed to therapeutic ineffectiveness received in the previous calendar year |  |  |
| CP7. Percentage of reports on medication errors reported in the previous year |  |  |
| CP8. Percentage of registered pharmaceutical companies having a functional pharmacovigilance system |  |  |
| CP9. Number of active surveillance activities initiated, ongoing or completed during the past five calendar years | 4.5. Number of active surveillance activities currently ongoing or carried out in the last five years | VL04.08: Active vigilance activities, as well as proactive monitoring programmes (when needed) have been developed and implemented. |
| **Signal and data management** |  |  |
|  | 3.2. Existence of a database for tracking pharmacovigilance activities |  |
| CO1. Number of signals detected in the past 5 years by the pharmacovigilance centre |  | VL05.02: Performance indicators for vigilance activities are established. |
| CO2. Number of regulatory actions taken in the preceding year as a consequence of national pharmacovigilance activities includes  CO2a: number of product label changes (variation);  CO2b: number of safety warnings on medicines to: (i) health professionals, (ii) general public;  CO2c: number of withdrawals of medicines;  CO2d: number of other restrictions on use of medicines |  | VL05.01: Vigilance information is used in timely manner to amend existing regulatory  decisions or to issue new regulatory decisions or actions. |
| CO3. Number of medicine-related hospital admissions per 1000 admissions |  |  |
| CO4. Number of medicine-related deaths per 1000 persons served by the hospital per year | 4.6. Percentage of patients in public health programs for whom drug-related adverse events were reported in the last year (disaggregated by type of adverse event, drug, severity, outcomes, and demographics)  4.7. Percentage of patients undergoing treatment within a public health program whose treatment was modified because of treatment failure or ADRs in the last year (disaggregated by treatment failure and ADRs) |  |
| CO5. Number of medicine-related deaths per 100 000 persons in the population |  |  |
| CO6. Average cost (US$) of treatment of medicine-related illness |  |  |
| CO7. Average duration (days) of medicine-related extension of hospital stay |  |  |
| CO8. Average cost (US$) of medicine-related hospitalization |  |  |
| **Existence of Drug Information center** |  |  |
|  | 2.3. Existence of a medicine information or pharmacovigilance service that provides ADR and drug safety–related question-and-answer services |  |
| **Existence of Procedures, SOPs, Protocols** |  |  |
|  | 2.7. Existence of national pharmacovigilance guidelines updated within the last five years  2.8. Existence of protocols or SOPs for improving patient safety relating to medicine use | VL04.03: Standard procedures exist and are implemented for enforcement of the national vigilance system. |
|  | 2.14. Platform or strategy exists for the coordination of pharmacovigilance activities at the national level |  |
|  | 5.8. Percentage of the sampled Drug and Therapeutics Committees that have carried out pharmacovigilance activities or addressed medicine safety issues in the last year |  |
|  |  | VL04.05: Staff access to information resources relevant to vigilance processes (e.g., safety information sources and reference materials) is ensured. |
| **Sampling of Medicines** |  |  |
|  | 5.10. Percentage of medicines sampled in the last year that passed product quality tests |  |
| **Communication** |  |  |
|  | 2.9. Existence of a minimum core list of communication technologies to improve access to safety reporting and provision of medicine information  5.7 Average time lag between identification of safety signal of a serious ADR or significant medicine safety issue and communication to health care workers and the public | VL02.02: Documented procedures and mechanisms are implemented to ensure the involvement, coordination and communication among all stakeholders relevant to vigilance activities  VL06.02: Mechanism for regular feedback to all stakeholders on vigilance events exists and is complemented with a risk communication plan.  VL06.03: Vigilance data and findings are shared with relevant regional and international partners. |
