# Supplementary material for: A comparative analysis of three pharmacovigilance system assessment tools
Source: PLoS One. 2025 Jul 8;20(7):e0327363. doi: 10.1371/journal.pone.0327363 (PMC12237061; doi:10.1371/journal.pone.0327363)
Supplement: S2 Table — (DOCX) [file pone.0327363.s002.docx]

| **S2 Table:** Supplementary or complementary indicators for IPAT and WHO indicators | |
| --- | --- |
| **(Indicator Code) WHO Complementary indicators** | **(Indicator No.) IPAT Supplementary indicators** |
| **Legal Provisions Regulations and Guidelines** |  |
| ST11. Existence of requirements mandating market authorization holders to submit periodic safety update reports | - 1. Legal provisions require that the marketing authorization holder mandatorily report all serious ADRs to the national drug regulatory authority   2. Legal provisions require the marketing authorization holder to conduct the same or similar postmarketing surveillance activities for products as required by |
| **Existence of communication technologies/ System/ reference material** |  |
| ST1. Existence of a dedicated computer for pharmacovigilance activities  ST3. Existence of functioning and accessible communication facilities in the pharmacovigilance centre  ST2. Existence of a source of data on consumption and prescription of medicines  ST4. Existence of a library or other reference source for drug safety information  ST5. Existence of a computerized case-report management system  ST6. Existence of a programme (including a laboratory) for monitoring the quality of pharmaceutical products  *Subset indicator* ST6a: The programme (including a laboratory) for monitoring the quality of pharmaceutical products collaborates with the pharmacovigilance programme  ST7. Existence of an essential medicines list which is in use  ST8. Systematic consideration of pharmacovigilance data when developing the main standard treatment guidelines | 2.11. Percentage of predefined core reference materials available in the medicine information or pharmacovigilance center |
| **Training of HCPs** |  |
| ST9. The pharmacovigilance centre organizes training courses  ST9a: for health professionals;  ST9b: for the general public  ST10. Availability of web-based pharmacovigilance training tools ST10a: for health professionals;  ST10b: for the general public | 2.13. Number of health care providers trained on pharmacovigilance and medicine safety in the last year |
| Risk Assessment and evaluation |  |
| P1. Percentage of health-care facilities with a functional pharmacovigilance unit (i.e. submitting ≥ 10 reports to the pharmacovigilance centre) in the previous year  P2. Percentage of total reports sent in the previous year by the different stakeholders includes  P2a: percentage of total reports sent by medical doctors;  P2b: by dentists;  P2c: by pharmacists;  P2d: by nurses or midwives;  P2e: by the general public;  P2f: by manufacturers  P3. Total number of reports received per million population per year  P4. Average number of reports per number of health-care providers per year includes  P4a: by medical doctors;  P4b: by dentists;  P4c: by pharmacists;  P4d: by nurses or midwives  P5. Percentage of health-care providers aware of and knowledgeable about ADRs per facility  P6. Percentage of patients leaving a health facility aware of ADRs in general  P7. Number of face-to-face training sessions in pharmacovigilance organized in the previous year  P7a: for health professionals;  P7b: for the general public  P8. Number of individuals who received face-to-face training in pharmacovigilance in the previous year  P8a: number of health professionals trained in the previous year;  P8b: number of individuals from the general public trained in the previous year  P9. Total number of national reports for a specific product per volume of sales of that product in the country (product specific) from the industry  P10. Number of registered products with a pharmacovigilance plan and/or a risk management strategy among the marketing authorization holders in the country  *Subset indicator* P10a: Percentage of registered products with a pharmacovigilance plan and/or a risk management strategy from the market authorization holders in the country  P11. Percentage of market authorization holders who submit periodic safety update reports to the regulatory authority as stipulated in the country  P12. Number of products voluntarily withdrawn by market authorization holders because of safety concerns in the previous year  *Subset indicator* P12a: Number of summaries of product characteristics (SPCs) updated by market authorization holders because of safety concerns in the previous year  P13. Number of reports from each registered pharmaceutical company received by the pharmacovigilance centre in the previous year | 4.1. Number of medicine utilization reviews carried out in the last year  4.3. Incidence of medication errors quantified in the last year  4.8. Percentage of patients in public health programs for whom drug-related, serious “unexpected adverse events” were reported in the last year  5.2. Prequalification schemes (e.g., WHO prequalification program and Pharmaceutical Inspection Co-operation Scheme) used in medicine procurement decisions  5.3. in the last year, medicine safety information requests received and addressed |
| **Risk mitigation and Impact** |  |
| O1. Percentage of preventable ADRs reported in the previous year out of the total number of ADRs reported  O2. Number of medicines-related congenital malformations per 100 000 births  O3. Number of medicines found to be possibly associated with congenital malformations in the past 5 years  O4. Percentage of medicines in the pharmaceutical market that are counterfeit/substandard  O5. Number of patients affected by a medication error in hospital per 1000 admissions in the previous year  O6. Average work or schooldays lost due to drug-related problems  O7. Cost savings (US$) attributed to pharmacovigilance activities  O8. Health budget impact (annual and over time) attributed to pharmacovigilance activity  *Rational use of medicines*  O9. Average number of medicines per prescription  O10. Percentage of prescriptions with medicines exceeding manufacturer’s recommended dose  O11. Percentage of prescription forms prescribing medicines with potential for interaction  O12. Percentage of patients receiving information on the use of their medicines and on potential ADRs associated with those medicines | 5.1. Risk mitigation plans currently in place that are targeted at high-risk medicines |
