# Supplementary material for: A comparative analysis of three pharmacovigilance system assessment tools
Source: PLoS One. 2025 Jul 8;20(7):e0327363. doi: 10.1371/journal.pone.0327363 (PMC12237061; doi:10.1371/journal.pone.0327363)
Supplement: S3 Table — (DOCX) [file pone.0327363.s003.docx]

| **S3 Table. Indicators for PHPs** | |
| --- | --- |
| **(Indicator No.) IPAT indicators for PHPs** | **(Indicator Code) WHO indicators for PHP** |
| 1.1, 2.1, 2.2, 2.3, 2.4, 2.5, 2.8, 2.9, 2.10, *2.11, 2.13,* 3.3, 3.4, 3.5, 3.6, *4.1, 4.3*, 4.4, 4.5, 4.6, 4.7, *4.8, 5.1, 5.2, 5.3, 5.4, 5.5, 5.6,* 5.7*, 5.9,* 5.10 | PH1. PH2. PH3. PH4. PH5. PH6. PH7. PH8. PH9. |
